# Palladium-Functionalized Polysiloxane Drop-Casted on Carbon Paper as a Heterogeneous Catalyst for the Suzuki–Miyaura Reaction

**DOI:** 10.3390/polym16192826

**Published:** 2024-10-06

**Authors:** Ekaterina A. Golovenko, Anastasia N. Kocheva, Artem V. Semenov, Svetlana O. Baykova, Konstantin V. Deriabin, Sergey V. Baykov, Vadim P. Boyarskiy, Regina M. Islamova

**Affiliations:** Institute of Chemistry, St. Petersburg State University, 7/9 Universitetskaya nab., 199034 St. Petersburg, Russia; st096793@student.spbu.ru (E.A.G.); st110199@student.spbu.ru (A.N.K.); a.v.semenov@spbu.ru (A.V.S.); s.baykova@spbu.ru (S.O.B.); st017488@student.spbu.ru (K.V.D.); sergei.v.baikov@yandex.ru (S.V.B.)

**Keywords:** palladium(II) complexes, polysiloxanes, macroheterogeneous catalysts, Suzuki–Miyaura reaction

## Abstract

In this work, a Pd(II)-*C,N*-cyclometalated complex was grafted to polysiloxanes via azide–alkyne cycloaddition. The obtained polymer–metal complex (Pd-PDMS) acts as a catalyst in the Suzuki–Miyaura reaction. Pd-PDMS was drop-casted onto a carbon fiber support, and the resulting membrane demonstrated catalytic activity in the cross-coupling reaction without yield loss after several catalytic cycles. The catalytic membrane allows for easy catalyst recycling and provides ultra-low palladium levels in Suzuki–Miyaura reaction products.

## 1. Introduction

The Suzuki–Miyaura (SM) reaction is extensively used for industrial synthesis of different pharmaceutically active compounds, such as benzodiazepines, angiotensin receptor blockers, non-steroidal anti-inflammatory drugs, kinase inhibitors, protease inhibitors, etc. [1]. The SM reaction represents carbon–carbon cross-coupling between a wide range of aryl halides and arylboronic acids in a different media, usually in the presence of palladium complexes with various ligands [2] including phosphine-based ones and N-based ligands such as Schiff bases, amines, oximes, hydrazones and N-heterocyclic carbenes [3]. There have been many successful demonstrations of homogeneous catalysis in SM reactions [4,5]. The advantages of homogeneous catalysis include high catalyst activity and short reaction times. The main problem with the use of homogeneous catalysis is the metal contamination of pharmaceutically active compounds. Thus, in accordance with the European Medicines Agency, the Pd contents for oral and parenteral medicines should be less than 10 and 1 ppm, respectively [6]. In order to reduce the metal content in the products of SM coupling reactions after homogeneous catalysis, lots of strategies have been proposed, such as crystallization, treatment by solvents with different polarities, and nanofiltration [7]. All the procedures increase costs and do not allow for the complete removal of Pd-containing residue from the target product [8,9]. Moreover, homogeneous catalysis usage creates shortcomings in precious metal containing catalyst recycling and reuse [10].

An alternative to homogeneous catalysis is heterogeneous catalysis [11]. In spite of some disadvantages, such as lower reaction rates and yields, heterogeneous catalysts are easier to recover and reuse several times [12]. Moreover, use of a heterogeneous catalyst on different supports allows us to reduce the metal content in the target product, which is crucial for biomedical applications [13,14]. The supports for heterogeneous catalysts include metal–organic frameworks [15], magnetic supports [16], carbon [17] and mesoporous silica [18]. However, activated carbons and metal–organic frameworks demonstrate low stability in air and moisture [19], and magnetic nanoparticles lack structural variability and have a tendency to aggregate [20]. The disadvantage of mesoporous silica is the amorphous pore walls and the high concentration of silanol groups on the surface [21], which can lead to the catalysis of side reactions and, as a consequence, to a drop in the selectivity of the catalyst [22]. Moreover, the above-mentioned supports do not demonstrate film-forming properties and require centrifugation for their separation and reuse.

The rapid development of polymer chemistry led to them becoming promising catalyst carriers. Polymers possess an adjustable solubility and air and moisture stability, and some of them are thermally stable and demonstrate good film-forming properties. One of the most widely used polymer supports (matrix) for Pd-containing heterogeneous catalysts is polystyrene [23]. In ref. [24], styrene was copolymerized with maleic anhydride; the obtained polymer was modified by 2-aminothiazole, and then PdCl_2_ was coordinated to the preprepared support. The main problem was palladium leaching from the polymer materials, which restricts the application of the resulting heterogeneous catalysts. Recently, the immobilization of Pd(II) with a pyridine-2,6-bisamide ligand on a polystyrene matrix was proposed [25]. The obtained catalysts were used in the form of powder.

Alternatively, polysiloxanes can be used. Polysiloxanes demonstrate outstanding film-forming properties and can be cross-linked or used as a film applied to different surfaces. Furthermore, they are thermally and UV-stable, bioinert, flexible and have a low glass transition temperature (−125 °C) [26]. Polysiloxanes are insoluble in water and alcohols, which makes them promising matrixes for Pd-containing catalysts for SM reactions. Previously, PdCl_2_(CH_3_CN)_2_ was coordinated to amino-containing polysiloxanes, and this insoluble complex was used in SM reactions [27]. In a similar study, Pd(OAc)_2_ was supported on triazolyl-functionalized polysiloxanes [28]. The obtained complex was also insoluble in organic solvents. This creates limitations in regard to the application of the obtained polymer–metal complex as a film for subsequent use in heterogeneous catalysis in the form of, e.g., a catalytic membrane [29] which can be easily removed from the reaction mixture and reused. There are many examples of Pd-supported catalysts [30] that allow for obtaining SM-coupling products (some of them are presented in Table 1), but none of them was presented in the form of a film. Additionally, none of these studies reported whether immobilization produced a palladium-free product.

In our previous study, we successfully synthesized Pt(II)-containing polysiloxane via an azide–alkyne cycloaddition (CuAAC) reaction between Pt(II)-cyclometalated complexes and poly((3-azidopropyl)methylsiloxane-*co*-dimethylsiloxane) [34]. The obtained polymer–metal complex demonstrated good film-forming properties and catalytic performance in Si–O dehydrocoupling reactions. A similar strategy can be utilized for polysiloxane modification with a Pd(II)-cyclometalated complex in order to obtain a novel heterogeneous catalyst for SM cross-coupling reactions.

Thus, the objectives of the study are (*i*) to synthesize Pd(II)-containing polysiloxanes via CuAAC reaction; (*ii*) to characterize the synthesized polymer–metal complex by NMR spectroscopy; (*iii)* to determine a catalytic activity of the obtained polymer–metal complex in SM cross-coupling reactions and to control the level of palladium in the product of the reaction by ICP-AES.

## 2. Materials and Methods

### 2.1. Materials

Phenylboronic acid (95%), 4-bromotoluene (98%), and [Cu(CH_3_CN)_4_]PF_6_ (97%) were purchased from Merck KGaA (St. Louis, MO, USA). Triethanolamine (98%) was supplied from ABCR GmbH (Karlsruhe, Germany). Anhydrous Na_2_SO_4_ (99%), K_2_CO_3_ (99%), Na_2_EDTA (99%), ethyl acetate (99%), CHCl_3_ (99%), CH_2_Cl_2,_ (99%) CH_3_OH (99%), *i*-PrOH (99%) and CH_3_CN (99%) were purchased from Vekton (St. Petersburg, Russia) and used as received. Concentrated HCl (*ρ* = 1.2 g∙mL^−1^) and concentrated HNO_3_ (*ρ* = 1.4 g∙mL^−1^) were obtained from Vekton (St. Petersburg, Russia). *cis*-[PdCl_2_(CNXyl)_2_] and 2-(2-(3-methoxy-4-(prop-2-yn-1-yloxy)benzylidene)hydrazineyl)pyridine (MPBHP) were obtained according to the synthetic procedures reported in refs. [34,35]. Poly((3-azidopropyl)methylsiloxane-*co*-dimethylsiloxane) (N_3_-PDMS) containing 25 mol.% of (3-azidopropyl)methylsiloxane units was synthesized according to the previously published method [36]. The number-average molecular weight of N_3_-PDMS was equal to *M_n_* = 14,600 (dispersity *Đ* = 1.50) [36].

### 2.2. Methods

Nuclear magnetic resonance (NMR) spectroscopy was performed on the Bruker Avance III 400 NMR spectrometer in chloroform-*d* and methanol-*d_4_* at room temperature (RT, 23 °C) and 30 °C (operating at 400 MHz for ^1^H, 100 MHz for ^13^C{^1^H}). Chemical shifts of signals are reported in *δ*-values [ppm] relative to the residual signals of solvent peaks for chloroform-*d*: *δ* = 7.28 (^1^H), 77.2 (^13^C{^1^H}); for methanol-*d_4_*: *δ* = 4.78 and 3.31 (^1^H), 49.2 (^13^C{^1^H}). The ^13^C{^1^H} NMR spectra were recorded with ^1^H decoupling. The following abbreviations were utilized to designate multiplicities: s = singlet, d = doublet, t = triplet, m = multiplet, br. = broad, dd = doublet of doublets, ddd = doublet of doublet of doublets.

High-resolution electrospray ionization (HRESI^+^) mass spectrometry. The HRESI^+^ mass spectra were registered on a Shimadzu Nexera X2 LCMS-9030 (Shimadzu, Kyoto, Japan) spectrometer equipped with an electrospray ionization source. The analyzed samples were dissolved in pure CH_3_OH prior to the HRESI^+^ mass spectrometric measurements. The equipment was operated in a positive ion mode using an *m/z* range of 50–3000. The most intensive peak in the isotopic pattern was noted.

X-ray photoelectron spectroscopy (XPS) was carried out on an Escalab 250Xi photoelectron spectrometer (ThermoScientific, Waltham, MA, USA) with AlK_α_ radiation (photon energy 1486.6 eV). The spectra were recorded in the constant pass energy mode at 100 eV for survey spectrum and 50 eV for the element core level spectrum using an XPS spot size of 650 μm.

The energy-dispersive X-ray (EDX) spectroscopy was acquired at the EDXRF spectrometer Shimadzu EDX-800P (Shimadzu, Japan) with Oxford Instruments XTF-5011A X-ray tube (Rh target, power 50 W).

The Raman spectra were obtained through backscattering geometry at the Raman spectrometer Senterra (Bruker, Bremen, Germany) equipped with a 532 nm wavelength solid-state laser (the corresponding energy is 2.33 eV). The laser power under ×20 (numerical aperture = 0.4) objective was approximately 66 µW (*λ* = 532 nm). The diffraction grading has 400 lines per mm, and the aperture was 25 × 1000 µm.

Scanning electron microscopy (SEM) images of the samples’ surfaces were obtained at RT on a Zeiss Auriga Crossbeam system (Carl Zeiss, Jena, Germany) with accelerating voltages ranging from 5 to 20 kV and working distances ranging from 8.7 to 8.9 mm.

ICP-AES. The palladium content in the product of SM reaction was determined by ICP-AES. Before the analysis, the sample was refluxed in 5.0 mL of hydrochloric acid and the nitric acid mixture (3:1, *v*/*v*). After cooling, the sample was quantitatively transferred to a volumetric flask and adjusted with a solution of 0.1 N HNO_3_ to 50 mL. Then, an ICP-AES analysis was conducted on a Shimadzu ICPE-9000 spectrometer. The palladium concentration was determined at *λ* = 367.47 nm. Standard samples of analyzed elements were prepared from a multi-component standard (Merck, St. Louis, MO, USA) in 0.1 N HNO_3_ for a calibration curve (0.001–10 mg·dm^−3^).

### 2.3. Synthesis of Palladium(II) C,N-Cyclometalated Complex (CMP)

**Scheme 1:**
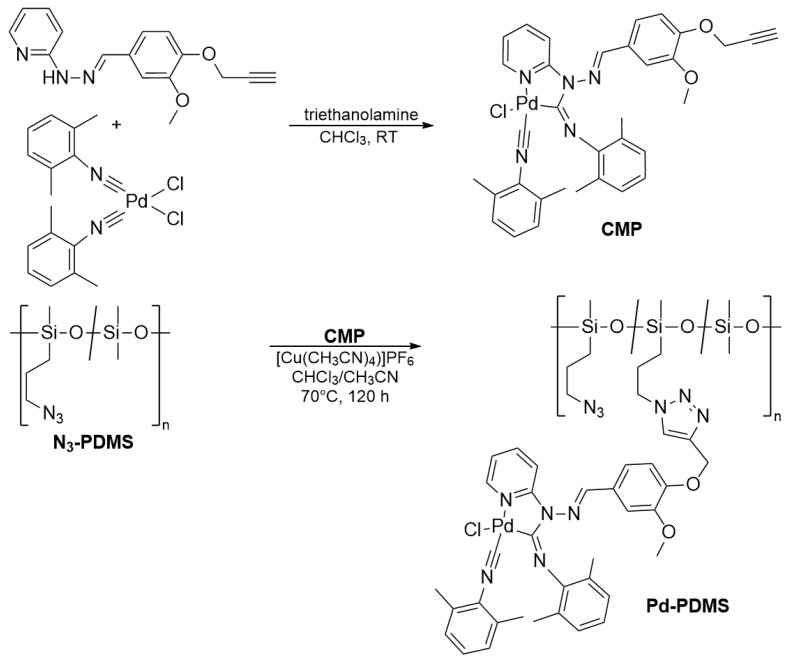
Synthesis of Pd-PDMS.

*cis*-[PdCl_2_(CNXyl)_2_] (800 mg, 1.82 mmol) and MPBHP (512 mg, 1.82 mmol) were dissolved in CHCl_3_ (40 mL), and then triethanolamine (271 mg, 1.82 mmol) was added to the obtained solution (Scheme 1). The reaction mixture was stirred for 72 h at RT. The formed precipitate was filtered off, while much of the CHCl_3_ was evaporated from supernatant under reduced pressure (down to 5 mbar). Afterwards, 30 mL of CH_3_OH was added to the resulting solution, forming a precipitate, which was filtered and dried. Yield: 984 mg (79%); pale yellow powder. ^1^H NMR (chloroform-*d*, *δ*, ppm): 9.66 (s, 1H, *l*-H), 9.14 (dd, *J_1_* = 5.9 Hz, *J_2_* = 1.6 Hz, 1H, *h*-H), 7.84 (ddd, *J_1_* = 7.9 Hz, *J_2_* = 1.6 Hz, 1H, *f*-H), 7.49 (d, *J* = 1.6 Hz, 1H, *i*-H), 7.41 (d, *J* = 7.9 Hz, 1H, *e*-H), 7.25 (dd, *J_2_* = 1.6 Hz, 1H, *k*-H), 7.13 (t, *J* = 7.6 Hz, 1H, *q*-H), 7.07 (d, *J* = 8.3 Hz, 1H, *j*-H), 7.01–6.93 (m, 3H, *p*-H + *g*-H), 6.78 (d, *J* = 7.5 Hz, 2H, *s*-H), 6.25 (t, *J* = 7.5 Hz, 1H, *t*-H), 4.82 (d, *J* = 2.3 Hz, 2H, *n*-H), 3.99 (s, 3H, *m*-H), 2.53 (t, *J* = 2.3 Hz, 1H, *o*-H), 2.27 (s, 6H, *r*-H), 2.24 (s, 6H, *u*-H) (Figure S1). ^13^C{^1^H} NMR (chloroform-*d*, *δ*): 157.7 (*l*-C), 158.6 (*w*-C), 158.5 (*q*-C), 150.5 (*i*-C), 150.0 (*h*-C), 149.4 (*f*-C), 147.1 (*e*-C), 140.8 (*k*-C), 134.2 (*s*-C), 129.0 (*r′*-C), 128.5 (*u*-C), 128.1 (*r*-C), 127.7 (*t′*-C), 127.5 (*d*-C), 123.6 (*u′*-C), 122.6 (*j*-C), 116.4 (*t*-C), 113.5 (*c*-C), 111.4 (*b*-C), 109.2 (*g*-C), 78.0 (*o*-C), 76.3 (*p*-C), 56.7 (*n*-C), 56.1 (*m*-C), 19.7 (*v*-C), 18.5 (*v′*-C) (Figure S2). HRESIMS^+^ *m/z* [M+H]^+^ calculated for [C_34_H_33_ClN_5_O_2_Pd]^+^ 684.1358; found 684.1357 (Figure S3). The XPS survey and Pd 3d core level spectra are shown in Figure S4.

### 2.4. Synthesis of Palladium(II) C,N-Cyclometalated Complex-Triazolyl-Containing Polysiloxane (Pd-PDMS)

**Figure 1:**
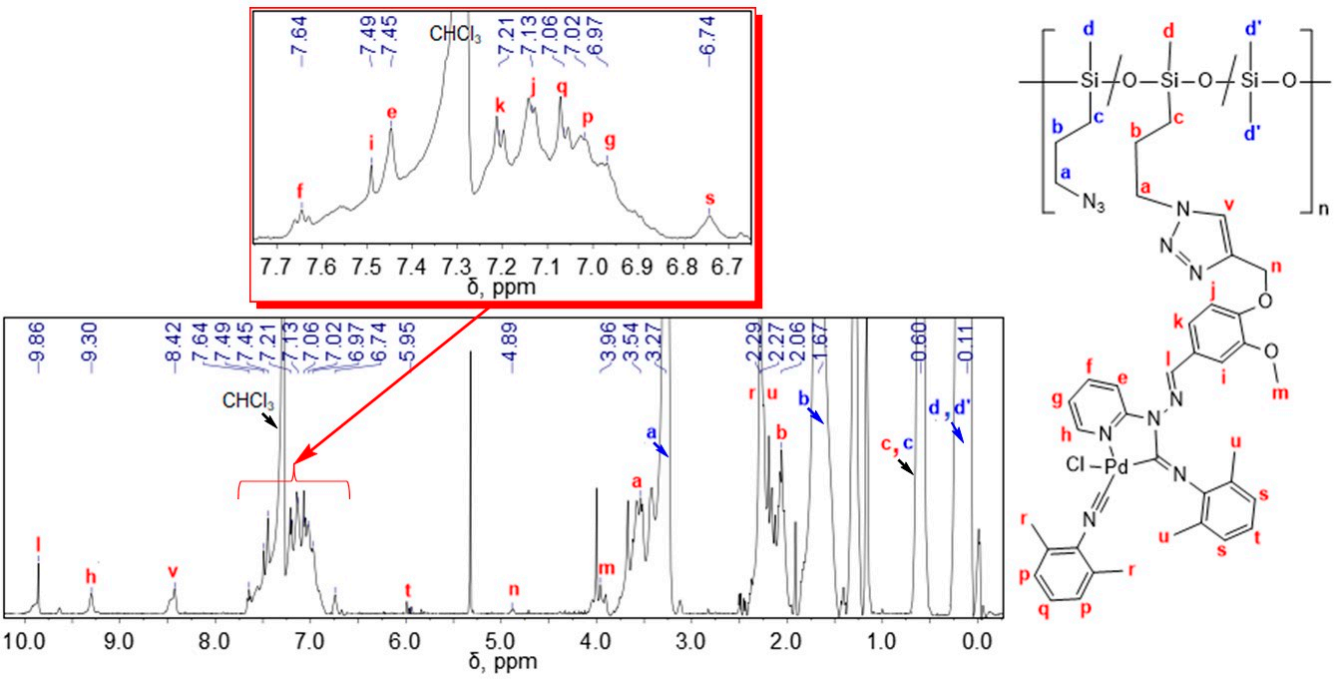
^1^H NMR of Pd-PDMS registered in CDCl_3_.

CMP (50.0 mg, 0.073 mmol), N_3_-PDMS (133.3 mg, 0.365 mmol of (3-azidopropyl)methylsiloxane units) and [Cu(CH_3_CN)_4_]PF_6_ (136.1 mg, 0.365 mmol) were dissolved in a CHCl_3_/CH_3_CN (4:1, *v*/*v*) mixture (20 mL). An obtained solution was then transferred to a flask in an argon atmosphere. The reaction was carried out at 70 °C for 120 h with vigorous stirring in an argon atmosphere. The color of the reaction mixture has changed from yellow to brown. After the completion of the reaction, the resulting solution was washed with a saturated aqueous Na_2_EDTA solution and then with distilled H_2_O. After that, the organic phase was dried over anhydrous Na_2_SO_4_ for 12 h and filtered. The solvent was removed by rotary evaporation under reduced pressure (down to 5 mbar, 50 °C). An obtained brown film of Pd-PDMS was dissolved in 50 mL of ethyl acetate and then centrifuged at 500 rpm for 5 min. The supernatant was collected and the ethyl acetate was removed by rotary evaporation (down to 5 mbar, 50 °C). The obtained purified polymer film of Pd-PDMS was further used in catalytic tests. Yield: 219 mg (57%). ^1^H NMR (chloroform-*d*, *δ*): 9.86 (br. s, *l*-H), 9.30 (br. m, *h*-H), 8.42 (br. s, *v*-H), 7.64 (br. t, *f*-H), 7.49 (br. s, *i*-H), 7.45 (br. d, *e*-H), 7.41 (br. m, *k*-H), 7.13 (br. m, *j*-H), 7.06 (br. m, *q*-H), 7.02 (br. m, *p*-H), 6.97 (br. m, *g*-H), 6.74 (br. m, *s*-H), 5.95 (br. m, *t*-H), 4.89 (m, *n*-H), 3.96 (s, *m*-H), 3.54 (br. m, SiCH_2_CH_2_C*H*_2_N), 3.27 (br. m, high intensity, SiCH_2_CH_2_C*H*_2_N_3_), 2.29 (br. s, *r*-H), 2.27 (br. s, *u*-H), 2.06 (br. m, SiCH_2_C*H*_2_CH_2_N), 1.67 (br. m, high intensity, SiCH_2_C*H*_2_CH_2_N_3_), 0.60 (br. m, SiC*H*_2_CH_2_CH_2_N), 0.1 (br. s, high intensity, SiC*H_3_*) (Figure 1).

### 2.5. Catalytic Tests

#### 2.5.1. Carrying Out SM Reaction with Pd-PDMS Applied to the Vial Wall (Solvent–Methanol-*d*_4_)

Pd-PDMS (2.1 mg) was dissolved in CH_2_Cl_2_ (0.15 mL) and placed in a 3 mL vial. CH_2_Cl_2_ was evaporated in vacuo (Figure S5a). After the catalytic film formation, phenylboronic acid (13.6 mg, 0.11 mmol), 4-bromotoluene (17.1 mg, 0.10 mmol), K_2_CO_3_ (20.7 mg, 0.15 mmol), and methanol-*d*_4_ (1 mL) were added to the vial. The reaction was performed at 90 °C for 6 h with stirring. In order to prevent the evaporation of 4-bromotoluene, the reaction was carried out in a sealed vial. The vial was weighted before and after the reaction (no weight loss was detected). After completion of the reaction, an aliquot of the reaction mixture (0.6 mL) was transferred in an NMR tube and the ^1^H NMR spectrum was registered at 30 °C. The yield of the target product 4-methylbiphenyl was calculated in the ^1^H NMR spectrum from the ratio of the intensities of the peaks corresponding to the methyl (CH_3_–) group of 4-methylbiphenyl and the CH_3_– group of the starting 4-bromotoluene.

#### 2.5.2. Carrying Out SM Reaction with Pd-PDMS Applied to Carbon Paper (CP) (Solvent–Methanol-*d*_4_)

Pd-PDMS (2.1 mg) was dissolved in CH_2_Cl_2_ (0.15 mL). The obtained solution was drop-casted to a piece of CP (1 cm^2^). A piece of CP with the applied catalyst was placed in a vial (Figure S5b). SEM images of Pd-PDMS applied on CP are presented in Figure S6a, while details on CP characterization are featured in Section S3 of SI. Then, phenylboronic acid (13.6 mg, 0.11 mmol), 4-bromotoluene (17.1 mg, 0.10 mmol), K_2_CO_3_ (20.7 mg, 0.15 mmol), and methanol-*d*_4_ (1.0 mL) were added to the vial. The reaction was performed at 90 °C for 6 h with continuous stirring. In order to prevent the evaporation of 4-bromotoluene, the reaction carried out in a sealed vial. The vial was weighted before and after the reaction (no weight loss was detected). After completion of the reaction, an aliquot of the reaction mixture (0.6 mL) was transferred in an NMR tube and the ^1^H NMR spectrum was registered at 30 °C. The yield of the target product 4-methylbiphenyl was calculated in the ^1^H NMR spectrum from the ratio of the intensities of the peaks corresponding to the methyl (CH_3_–) group of 4-methylbiphenyl and the CH_3_– group of the starting 4-bromotoluene.

#### 2.5.3. Carrying Out SM Reaction with Pd-PDMS Applied to CP (Solvent–Methanol-d_4_:D_2_O in a *v*/*v* Ratio 4:1)

Pd-PDMS (2.1 mg) was dissolved in CH_2_Cl_2_ (0.15 mL). The obtained solution was drop-casted to a piece of CP (1 cm^2^). A piece of CP with the applied catalyst was placed in a vial (Figure S5b). SEM images of Pd-PDMS applied on CP are presented in Figure S6b,c. Then, phenylboronic acid (13.6 mg, 0.11 mmol), 4-bromotoluene (17.1 mg, 0.10 mmol), K_2_CO_3_ (20.7 mg, 0.15 mmol), methanol-*d*_4_ (0.8 mL of) and D_2_O (0.2 mL) were added to the vial. The reaction was performed at 90 °C for 6 h with continuous stirring. In order to prevent the evaporation of 4-bromotoluene, the reaction was carried out in a sealed vial. The vial was weighted before and after the reaction (no weight loss was detected). After completion of the reaction, an aliquot of the reaction mixture (0.6 mL) was transferred in an NMR tube and the ^1^H NMR spectrum was registered at 30 °C. The yield of the target product 4-methylbiphenyl was calculated in the ^1^H NMR spectrum from the ratio of the intensities of the peaks corresponding to the methyl (CH_3_–) group of 4-methylbiphenyl and the CH_3_– group of the starting 4-bromotoluene.

## 3. Results and Discussion

### 3.1. Synthesis of a Palladium(II) C,N-Cyclometalated Complex and Azide–Alkyne Cycloaddition between the CMP and Azido-Containing Polysiloxanes

A new palladium(II) C,N-cyclometalated complex (CMP) was synthesized in a 79% yield by the nucleophilic addition reaction of MPBHP to the coordinated isocyanide ligand in the palladium(II) *bis*(isocyanide) complex *cis*-[PdCl_2_(CNXyl)_2_]. The reaction was carried out in CHCl_3_ at RT in the presence of triethanolamine as a base (Scheme 1). The obtained CMP was characterized by HRESI^+^ mass spectrometry, ^1^H, ^13^C{^1^H} NMR spectroscopy, and XPS. Afterwards, the CMP was grafted to poly(3-azidopropylmethylsiloxane-*co*-dimethylsiloxane) bearing 25% of N_3_-moieties via the azide–alkyne cycloaddition (CuAAC) reaction according to the slightly modified method reported in ref. [34] (Scheme 1).

The CuAAC reaction was performed in the presence of the [Cu(CH_3_CN)_4_]PF_6_ catalyst in a mixture of solvents CHCl_3_:CH_3_CN in a ratio of 4:1 (*v*/*v*) due to the solubility of the CMP and polysiloxanes in organochlorine solvents and the solubility of the Cu(I) complex in CH_3_CN. By analogy with ref. [34], the molar ratio of the CMP and N_3_-group of polysiloxanes was 1 to 5. The reaction was performed at 70 °C for 120 h under vigorous stirring. The obtained polymer (Pd-PDMS) was purified by being dissolved in ethylacetate and subsequent centrifugation to separate the unreacted CMP. The supernatant after centrifugation was collected and the solvent was evaporated under reduced pressure (down to 5 mbar) on a rotary evaporator. The obtained Pd-PDMS presented a dark brown film and, when freshly prepared, was soluble in organochlorine solvents, benzene and ethyl acetate but was insoluble in alcohols. Due to the solubility of freshly prepared Pd-PDMS in chloroform, its structure was defined using NMR spectroscopy. The ^1^H NMR spectrum of the obtained Pd-PDMS is presented in Figure 1.

In the ^1^H NMR spectrum of Pd-PDMS, signals corresponding to the protons of the CMP were detected at 9.86, 9.30, 7.64, 7.49, 7.45, 7.21, 7.13, 7.06, 7.03, 6.97, 6.74 5.95, 4.89, 3.96, 2.29 and 2.27 ppm. No presence of the ethynyl group of the initial CMP was found. The signal of the hydrogen atom of the formed triazole ring resulting from the azide–alkyne cycloaddition of the CMP to N_3_-PDMS is detected at 8.42 ppm. The amount of reacted azide groups was estimated from the ^1^H NMR data and is equal to 5%, which is close to ref. [34].

**Figure 2:**
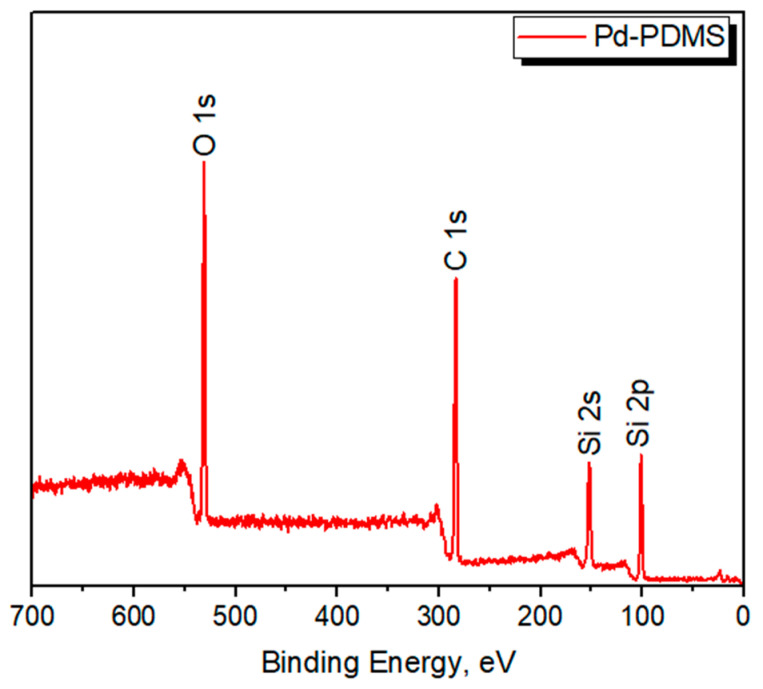
XPS spectrum of Pd-PDMS.

The obtained Pd-PDMS was also analyzed by XPS. Prior to analysis, Pd-PDMS was dissolved in CH_2_Cl_2_ and drop-casted to CP. The XPS spectrum is presented in Figure 2.

**Figure 3:**
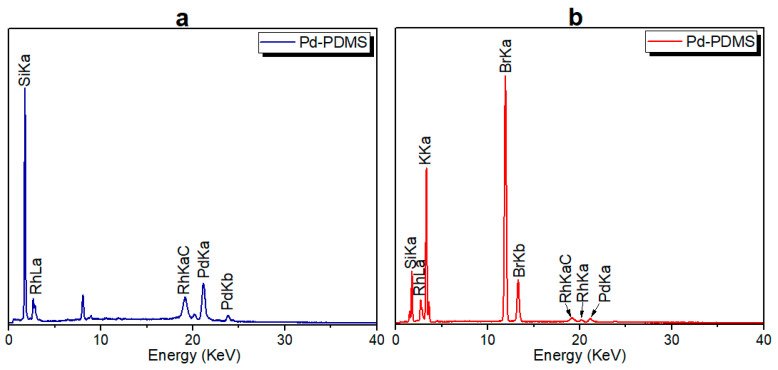
EDX spectra of Pd-PDMS before catalysis (**a**) and Pd-PDMS on CP after the third catalytic cycle (**b**).

In the XPS spectrum, elements forming a polysiloxane chain, such as silicon, carbon and oxygen, with a value of binding energy of ~101, 284, 531 eV for Si 2p, C 1s, O 1s, respectively, were detected. However, due to the possible non-uniform distribution of the CMP embedded in the polysiloxane matrix [37], as well as the sensitivity of XPS survey scans, which can be not high enough for certain species, Pd 3d with local maxima at binding energies of ~341 eV (Pd 3d_3/2_) and 337 eV (Pd 3d_5/2_) was not detected by this method. Therefore, we investigated the presence of Pd by energy-dispersive X-ray spectroscopy (EDX). The EDX spectra are presented in Figure 3. In the spectrum before catalytic cycles (Figure 3a), the presence of palladium and silicon was detected in a range of 0–40 KeV and the Pd content was estimated to be about 0.01 wt.%.

### 3.2. SM Reaction Catalyzed by Pd-PDMS

First of all, we tested the fundamental possibility of using the obtained palladium-containing polysiloxane as a catalyst for the SM cross-coupling. For this, we dissolved the required amount of Pd-PDMS (more details featured in 2.5 Catalytic tests) in CH_2_Cl_2_ and transferred the solution in a vial. After CH_2_Cl_2_ evaporation, we performed an SM reaction between 4-bromotoluene and phenylboronic acid in methanol-*d*_4_ in the presence of K_2_CO_3_ at 90 °C for 6 h with stirring. The reaction was carried out in a sealed vial to prevent evaporation 4-bromotoluene. After the completion of the reaction, the reaction mixture was analyzed by ^1^H NMR. The product yield was determined by comparing the ratio of the intensities of the peaks corresponding to the methyl (CH_3_–) group of the product (4-methylbiphenyl) and the (CH_3_–) group of the starting 4-bromotoluene. We found that the yield of 4-methylbiphenyl was 20%. Preliminary destruction of the film with a glass rod and subsequent sonication allowed us to reach a 30% yield of 4-methylbiphenyl. Thus, we have shown that the obtained polymer is capable of catalyzing the SM reaction; however, in concentrated form, its activity is insufficient.

Next, we tested our assumption about the possibility of obtaining a catalytic film based on the resulting polymer. For this, we used carbon paper (CP). For CP characterization, the Raman spectrum was acquired (the Raman spectrum is presented in Figure S8). In a first order spectrum there are two main bands, named D and G, with a maxima of 1363 and 1593 cm^−1^, respectively. The D-band usually results from the disorder-induced mode in carbon materials, while the G-band is connected with the tangential vibrations of carbon atoms of E_2*g*_ symmetry in graphene sheets [38]. There is an assembly of graphene sheets in the carbon matrix which forms carbon fibers [39]. Thus, the carbon fibers present a porous structure with a high surface-to-volume ratio. Additionally, the porosity of CP facilitates the diffusion of the reagents [40]. In our study, CP was chosen due to a physical adsorption of Pd-PDMS chains by CP driven by the interaction between (CH_3_–) groups of polysiloxanes and the π-electron-rich surface of carbon fibers [41]. To analyze CP used in catalytic experiments, the XPS spectrum was also acquired (Figure S8). The surface area of CP was calculated by the Brunauer–Emmett–Teller (BET) method using a nitrogen adsorption technique. The BET surface area is equal to 1.3942 m^2^∙g^−1^. Prior to Pd-PDMS deposition to CP, we sonicated CP in *i*-PrOH for 5 min, dried the CP and slowly drop-casted the required amount of Pd-PDMS in CH_2_Cl_2_ on the CP. As a result, we showed that palladium-containing polysiloxane can be deposited on the surface of CP to obtain a film/membrane (Figure S6).

**Figure 4:**
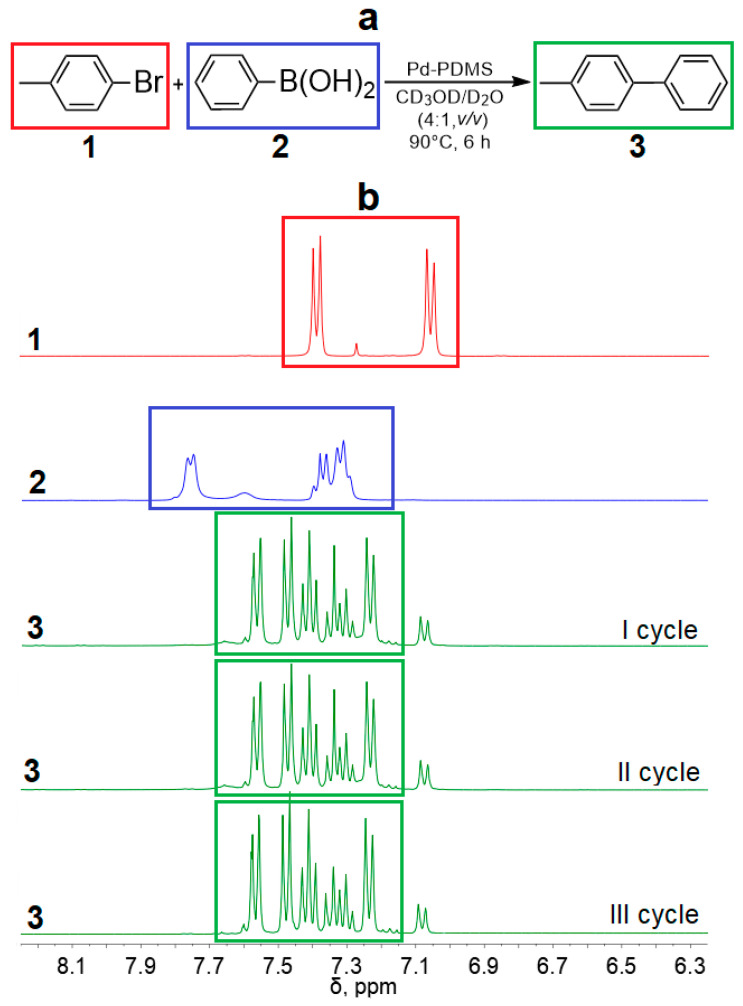
(**a**) SM reaction cross-coupling scheme; (**b**) ^1^H NMR spectra of initial 4-bromtoluene (1), phenylboronic acid (2), 4-methylbiphenyl (3) range from 6.25 to 8.25 ppm. The full range spectra are presented in Figure S7.

Afterwards, we tested the catalytic activity of the obtained membrane in the SM reaction. When the reaction was carried out in a methanol solution at 90 °C, a product yield of 55% was achieved in 6 h. However, when using a mixture of methanol-*d*_4_:D_2_O as a solvent, the yield of 4-methylbiphenyl under the same conditions increased to 80% (Figure 4).

The yields of the reactions under different conditions are given in Table 2.

The obtained data indicate that the reaction is preferable when carried out in a methanol-water mixture in a *v*/*v* ratio of 4:1 at 90 °C within 6 h. Carrying out the reaction in pure methanol showed worse results. This is probably due to the better sorption of the hydrophobic substrate (4-bromotoluene) on the surface of the catalytic film with a moderate increase in the water content in the solvent. Interestingly, in regard to the methanol-*d*_4_:D_2_O in *v*/*v* ratio (3:2), the yield of 4-methylbiphenyl significantly decreased to 30%, while, in regard to the methanol-*d*_4_:D_2_O in *v*/*v* ratio (2:3), the yield of 4-methylbiphenyl dropped to the even lower value of 10%. Previously, a strong dependence of the yield of SM cross-coupling reactions on water content in the reaction medium was demonstrated for the Pd(II) macrocyclic complex supported on polystyrene [25]. Increasing the temperature of the synthesis by up to 100 °C and the time of the SM reaction by up to 24 h did not affect the product yield (Table 2).

Use of CP allows us to not only increase the yield of 4-methylbiphenyl but also to remove the catalyst easily from the reaction mixture, pulling out the piece of CP with the applied Pd-PDMS from the vial. A purification of CP with Pd-PDMS after the reaction consists of short-washing with methanol at RT and short-drying. Then, CP with the applied Pd-PDMS is ready to use again. In contrast, heterogeneous catalysts in the form of powder require centrifugation, which can be difficult and unsuccessful, especially for viscous liquids in an industrial scale [42]. Interestingly, after the first catalytic cycle, Pd-PDMS becomes insoluble not only in alcohols but also in organochlorides, benzene, and toluene. This can be due to Pd(II) coordination with unreacted triazole rings [28].

Thus, using Pd-PDMS applied to CP, we conducted three catalytic cycles in SM reactions (Figure 4). After each cycle, the heterogeneous catalyst was easily removed from the reaction mixture, washed with methanol, dried, and placed back in the reaction mixture. No decrease in 4-methylbiphenyl yield was detected in each cycle (80 % in each cycle). When analyzing catalytic membrane by EDX (Pd-PDMS applied to CP), it also was found that, after the third catalytic cycle (Figure 3b), palladium and silicon in a ratio similar to the EDX spectrum of the initial Pd-PDMS (Figure 3a) are presented. Furthermore, potassium and bromine were detected in the spectra after the third catalytic cycle. This might be due the insolubility of the KBr formed during the SM reactions, as this which was not removed from the CP after its cleaning with methanol between catalytic cycles.

As we have already mentioned, for the practical application of the palladium catalyst in the synthesis of pharmaceutical substances, a low content of palladium in the reaction product is very important. Therefore, we tried to determine the concentration of palladium in the reaction mixture after the reaction. For this, we used the ICP-AES method. Our study showed that the amount of palladium in the studied solutions is below the detection limit of the method, 22 ppb, which is significantly lower than the palladium level required by European Medicines Agency [6]. This indicates an almost complete absence of palladium-leaching from the catalytic membrane and the potential applicability of our macroheterogeneous catalyst in the industrial synthesis of various active pharmaceutical ingredients (APIs).

## 4. Conclusions

We have shown that the cyclometalated palladium complex containing a propargyl group at the periphery of the ligand sphere can react with azido-modified polysiloxane. This results in a polysiloxane polymer with a firmly grafted palladium complex that is not removed by water or organic solvents. This polymer has catalytic activity in the SM reaction. Due to the properties of the polysiloxane carrier, it can disolve in organochlorine solvents and be applied to CP in this form, yielding a catalytic film with a developed surface. As a result, a catalytic membrane is formed that is resistant to leaching. This allows, firstly, for us to conveniently organize the catalyst recycling, which distinguishes the catalyst we proposed from other heterogeneous catalysts for the SM reaction described in the literature (Table 1). Secondly, this makes it possible to obtain a cross-coupling product that does not require purification from palladium. We believe that our approach will become popular in the industrial production of active pharmaceutical ingredients.

## Figures and Tables

**Table 1:** Heterogeneous palladium catalysts for the SM reaction.

Aryl Halyde *	Catalyst	Yield, % Fresh/After Recycling	Method of Catalyst Removing	Ref.
4′-Bromoaceto- phenone	Pd containing dioxime-functionalized Fe_3_O_4_	98/93	Not reported	[31]
4-Iodobenzene	Carbon nanotubes-supported Pd	94/96	Decantation	[32]
4-Iodotoluene	Pd supported on *Rosa* *canina* fruit extract	96/95	Filtration	[33]
4-Bromotoluene	Pd coordinated to amino-containing polysiloxanes	42	Filtration	[27]
2-Bromotoluene	Pd supported on triazolyl-functionalized polysiloxanes	99/29	Centrifugation	[28]

* in all reactions, phenylboronic acid was used as a coupling partner.

**Table 2:** The yield of 4-methylbiphenyl in an SM reaction between 4-bromotoluene and phenylboronic acid in the presence of K_2_CO_3_ with stirring (4-methylbiphenyl, phenylboronic acid and K_2_CO_3_ were taken with a molar ratio of 1:1.1:1.5; for more details, see 2.5 Catalytic tests).

Catalyst	Temperature of the Reaction, °C	Time of the Reaction, h	Solvent	Yield, %
Pd-PDMS applied to walls of a vial	90	6	methanol-*d*_4_	20
Crushed Pd-PDMS	80	6	methanol-*d*_4_	30
Crushed Pd-PDMS	90	6	methanol-*d*_4_	30
Pd-PDMS on CP	80	3	methanol-*d*_4_	45
Pd-PDMS on CP	80	6	methanol-*d*_4_	50
Pd-PDMS on CP	90	6	methanol-*d*_4_	55
Pd-PDMS on CP	90	6	methanol-*d*_4_:D_2_O in *v*/*v* ratio (10:1)	55
Pd-PDMS on CP	90	6	methanol-*d*_4_:D_2_O in *v*/*v* ratio (4:1)	80
Pd-PDMS on CP	90	24	methanol-*d*_4_:D_2_O in *v*/*v* ratio (4:1)	80
Pd-PDMS on CP	100	6	methanol-*d*_4_:D_2_O in *v*/*v* ratio (4:1)	80
Pd-PDMS on CP	90	6	methanol-*d*_4_: D_2_O in *v*/*v* ratio (3:2)	30
Pd-PDMS on CP	90	6	methanol-*d*_4_:D_2_O in *v*/*v* ratio (2:3)	10

## Data Availability

The data supporting this article have been included as a part of the Supplementary Materials.

## Supplementary Materials

The following supporting information can be downloaded at https://www.mdpi.com/article/10.3390/polym16192826/s1. Figure S1: ^1^H NMR of CMP registered in CDCl_3_; Figure S2: ^13^C{^1^H} NMR of CMP registered in CDCl_3_; Figure S3: HRESI^+^ mass spectrum of CMP complex; Figure S4: XPS survey spectrum (a) and Pd 3d core level spectrum (b) of CMP; Figure S5: Pd-PDMS applied on walls of a vial (a), Pd-PDMS on carbon paper (CP) (b); Figure S6: SEM images of pure CP (a), Pd-PDMS on CP before reaction (b) and Pd-PDMS on CP after reaction (c); Figure S7: ^1^H NMR spectra in a range from 1.75 to 8.25 ppm of 4-bromotoluene (1), phenylboronic acid registered in methanol-*d_4_* (2), methylbiphenyl 1st catalytic cycle (3.1), methylbiphenyl 2nd catalytic cycle (3.2), methylbiphenyl 3rd catalytic cycle (3.3); Figure S8: Spectra of the carbon paper used in in catalytic experiments: Raman (a) and XPS survey (b) spectra [43].

## References

[1] Farhang M., Akbarzadeh A.R., Rabbani M., Ghadiri A.M. (2022). A Retrospective-Prospective Review of Suzuki–Miyaura Reaction: From Cross-Coupling Reaction to Pharmaceutical Industry Applications. Polyhedron.

[2] Miyaura N., Suzuki A. (1995). Palladium-Catalyzed Cross-Coupling Reactions of Organoboron Compounds. Chem. Rev..

[3] Das P., Linert W. (2016). Schiff Base-Derived Homogeneous and Heterogeneous Palladium Catalysts for the Suzuki–Miyaura Reaction. Coord. Chem. Rev..

[4] Shen D., Xu Y., Shi S.-L. (2019). A Bulky Chiral N-Heterocyclic Carbene Palladium Catalyst Enables Highly Enantioselective Suzuki–Miyaura Cross-Coupling Reactions for the Synthesis of Biaryl Atropisomers. J. Am. Chem. Soc..

[5] Ashikari Y., Nagaki A. (2021). Homogeneous Catalyzed Aryl–Aryl Cross-Couplings in Flow. Synthesis.

[6] Pink C.J., Wong H., Ferreira F.C., Livingston A.G. (2008). Organic Solvent Nanofiltration and Adsorbents; A Hybrid Approach to Achieve Ultra Low Palladium Contamination of Post Coupling Reaction Products. Org. Process Res. Dev..

[7] Crawley M.L., Trost B.M. (2012). Applications of Transition Metal Catalysis in Drug Discovery and Development: An Industrial Perspective.

[8] Economidou M., Mistry N., Wheelhouse K.M.P., Lindsay D.M. (2023). Palladium Extraction Following Metal-Catalyzed Reactions: Recent Advances and Applications in the Pharmaceutical Industry. Org. Process Res. Dev..

[9] El Fadil A., Verbeke R., Kyburz M., Aerts P.E.M., Vankelecom I.F.J. (2023). From Academia to Industry: Success Criteria for Upscaling Nanofiltration Membranes for Water and Solvent Applications. J. Membr. Sci..

[10] Gao M., Wang J., Shang W., Chai Y., Dai W., Wu G., Guan N., Li L. (2023). Zeolite-Encaged Palladium Catalysts for Heterogeneous Suzuki-Miyaura Cross-Coupling Reactions. Catal. Today.

[11] Olmedo-Navarro M., Pérez J.M., Fernández I. (2024). Sustainable Synthesis and Characterization of Cobalt-Based Biocatalysts from Olive Stone Waste for Enhanced Curing of Unsaturated Polyester Resins. Polymer.

[12] Paul S., Clark J.H. (2003). A Highly Active and Reusable Heterogeneous Catalyst for the Suzuki Reaction: Synthesis of Biaryls and Polyaryls. Green Chem..

[13] Hajipour A.R., Shirdashtzade Z., Azizi G. (2014). Silica-Acac-Supported Palladium Nanoparticles as an Efficient and Reusable Heterogeneous Catalyst in the Suzuki–Miyaura Cross-Coupling Reaction in Water. J. Chem. Sci..

[14] Jo J., Tu Q., Xiang R., Li G., Zou L., Maloney K.M., Ren H., Newman J.A., Gong X., Bu X. (2019). Metal Speciation in Pharmaceutical Process Development: Case Studies and Process/Analytical Challenges for a Palladium-Catalyzed Cross-Coupling Reaction. Organometallics.

[15] Saha D., Sen R., Maity T., Koner S. (2013). Anchoring of Palladium onto Surface of Porous Metal–Organic Framework through Post-Synthesis Modification and Studies on Suzuki and Stille Coupling Reactions under Heterogeneous Condition. Langmuir.

[16] Zheng K., Shen C., Qiao J., Tong J., Jin J., Zhang P. (2018). Novel Magnetically-Recyclable, Nitrogen-Doped Fe_3_O_4_@Pd NPs for Suzuki–Miyaura Coupling and Their Application in the Synthesis of Crizotinib. Catalysts.

[17] Guillén E., Rico R., López-Romero J.M., Bedia J., Rosas J.M., Rodríguez-Mirasol J., Cordero T. (2009). Pd-Activated Carbon Catalysts for Hydrogenation and Suzuki Reactions. Appl. Catal. A Gen..

[18] Chen W., Li P., Wang L. (2011). Silica Supported Palladium-Phosphine Complex: Recyclable Catalyst for Suzuki–Miyaura Cross-Coupling Reactions at Ambient Temperature. Tetrahedron.

[19] Opanasenko M., Štěpnička P., Čejka J. (2014). Heterogeneous Pd Catalysts Supported on Silica Matrices. RSC Adv..

[20] Zhang Q., Yang X., Guan J. (2019). Applications of Magnetic Nanomaterials in Heterogeneous Catalysis. ACS Appl. Nano Mater..

[21] Liang J., Liang Z., Zou R., Zhao Y. (2017). Heterogeneous Catalysis in Zeolites, Mesoporous Silica, and Metal–Organic Frameworks. Adv. Mater..

[22] Björklund S., Kocherbitov V. (2017). Alcohols React with MCM-41 at Room Temperature and Chemically Modify Mesoporous Silica. Sci. Rep..

[23] Pittman C. (1976). 1,3-Butadiene Oligomerization Catalyzed by Polymer-Attached Palladium Complexes. Comparison with Homogeneous Catalysis. J. Catal..

[24] Heravi M.M., Hashemi E., Beheshtiha Y.S., Ahmadi S., Hosseinnejad T. (2014). PdCl 2 on Modified Poly (Styrene-Co-Maleic Anhydride): A Highly Active and Recyclable Catalyst for the Suzuki–Miyaura and Sonogashira Reactions. J. Mol. Catal. A Chem..

[25] Yamamoto K., Nameki R., Sogawa H., Takata T. (2020). Synthesis of Polystyrene-Supported Pd (II)-Containing Macrocyclic Complex as a Reusable Catalyst for Chemoselective Suzuki–Miyaura Coupling Reaction. Tetrahedron Lett..

[26] Mark J.E., Schaefer D.W., Lin G. (2015). The Polysiloxanes.

[27] Zawartka W., Pośpiech P., Cypryk M., Trzeciak A.M. (2015). Palladium Supported on Aminopropyl-Functionalized Polymethylsiloxane Microspheres: Simple and Effective Catalyst for the Suzuki–Miyaura C–C Coupling. J. Mol. Catal. A Chem..

[28] Mieczyńska E., Borkowski T., Cypryk M., Pospiech P., Trzeciak A.M. (2014). Palladium Supported on Triazolyl-Functionalized Polysiloxane as Recyclable Catalyst for Suzuki–Miyaura Cross-Coupling. Appl. Catal. A Gen..

[29] Domènech B., Muñoz M., Muraviev D.N., Macanás J. (2012). Catalytic Membranes with Palladium Nanoparticles: From Tailored Polymer to Catalytic Applications. Catal. Today.

[30] Mukai S., Yamada Y. (2022). Catalyst Recycling in the Suzuki Coupling Reaction: Toward a Greener Synthesis in the Pharmaceutical Industry. Knowledge.

[31] Kilic A., Gezer E., Durap F., Aydemir M., Baysal A. (2019). Pd (II) Supported Dioxime Functionalized Fe3O4 Nanoparticles as Efficient, Eco-Friendly and Reusable Catalysts for the Suzuki-Miyaura Cross-Coupling Reaction in Water. J. Organomet. Chem..

[32] Pan H., Yen C.H., Yoon B., Sato M., Wai C.M. (2006). Recyclable and Ligandless Suzuki Coupling Catalyzed by Carbon Nanotube-Supported Palladium Nanoparticles Synthesized in Supercritical Fluid. Synth. Commun..

[33] Veisi H., Rashtiani A., Barjasteh V. (2016). Biosynthesis of Palladium Nanoparticles Using *Rosa canina* Fruit Extract and Their Use as a Heterogeneous and Recyclable Catalyst for Suzuki–Miyaura Coupling Reactions in Water. Appl. Organomet. Chem..

[34] Deriabin K.V., Golovenko E.A., Antonov N., Baykov S., Boyarskiy V., Islamova R.M. (2023). Platinum-Macrocatalyst for Heterogeneous Si–O Dehydrocoupling. Dalton Trans..

[35] Mikherdov A.S., Kinzhalov M.A., Novikov A.S., Boyarskiy V.P., Boyarskaya I.A., Dar’in D.V., Starova G.L., Kukushkin V.Y. (2016). Difference in Energy between Two Distinct Types of Chalcogen Bonds Drives Regioisomerization of Binuclear (Diaminocarbene)Pd ^II^ Complexes. J. Am. Chem. Soc..

[36] Baranovskii E.M., Khistiaeva V.V., Deriabin K.V., Petrovskii S.K., Koshevoy I.O., Kolesnikov I.E., Grachova E.V., Islamova R.M. (2021). Re(I) Complexes as Backbone Substituents and Cross-Linking Agents for Hybrid Luminescent Polysiloxanes and Silicone Rubbers. Molecules.

[37] Shard A.G. (2014). Detection Limits in XPS for More than 6000 Binary Systems Using Al and Mg Kα X-rays. Surf. Interface Anal..

[38] Golovenko E.A., Pankin D.V., Deriabin K.V., Volkov A.I., Kirichenko S.O., Levin O.V., Islamova R.M. (2024). Ligand Exchange Reaction between Ferrocene and Multiwalled Carbon Nanotubes: A Contemporary Approach. Langmuir.

[39] Li Z., Deng L., Kinloch I.A., Young R.J. (2023). Raman Spectroscopy of Carbon Materials and Their Composites: Graphene, Nanotubes and Fibres. Prog. Mater. Sci..

[40] Torrinha Á., Martins M., Tavares M., Delerue-Matos C., Morais S. (2021). Carbon Paper as a Promising Sensing Material: Characterization and Electroanalysis of Ketoprofen in Wastewater and Fish. Talanta.

[41] Beigbeder A., Linares M., Devalckenaere M., Degée P., Claes M., Beljonne D., Lazzaroni R., Dubois P. (2008). CH-π Interactions as the Driving Force for Silicone-Based Nanocomposites with Exceptional Properties. Adv. Mater..

[42] Gandhi K., Sharma N., Gautam P.B., Sharma R., Mann B., Pandey V. (2022). Centrifugation. Advanced Analytical Techniques in Dairy Chemistry.

[43] Leadbeater N.E., Marco M. (2003). Transition-Metal-Free Suzuki-Type Coupling Reactions. Angew. Chem. Int. Ed..

