# Supplementary material for: Palladium-Functionalized Polysiloxane Drop-Casted on Carbon Paper as a Heterogeneous Catalyst for the Suzuki–Miyaura Reaction
Source: Polymers (Basel). 2024 Oct 6;16(19):2826. doi: 10.3390/polym16192826 (PMC11478592; doi:10.3390/polym16192826)
Supplement: Supplementary file 1 [file polymers-16-02826-s001.zip › polymers-3211097-supplementary.pdf]

Supporting Information

Palladium-functionalized polysiloxane drop-casted on carbon paper as a heterogeneous catalyst for Suzuki–Miyaura reaction

Ekaterina A. Golovenko, Anastasia N. Kocheva, Artem V. Semenov, Svetlana O. Baykova, Konstantin V. Deriabin, Sergey V. Baykov, Vadim P. Boyarskiy,* and Regina M. Islamova*

Institute of Chemistry, St. Petersburg State University, 7/9 Universitetskaya nab., St. Petersburg, 199034 Russia;

*Corresponding authors

E-mail address: v.boiarskii@spbu.ru (V.P.B.), r.islamova@spbu.ru (R.M.I.).

Contents

S1. Characterization of CMP and Pd-PDMS S2

S2. Catalytic performance of Pd-PDMS S4

S2.1. *Reaction conditions for Suzuki–Miyaura reaction in the presence of Pd-PDMS* S4

S2.2. *Suzuki–Miyaura cross-coupling between 4-bromotoluene and phenylboronic acid* .. S6

S3. Carbon paper characterization S8

S1. Characterization of CMP

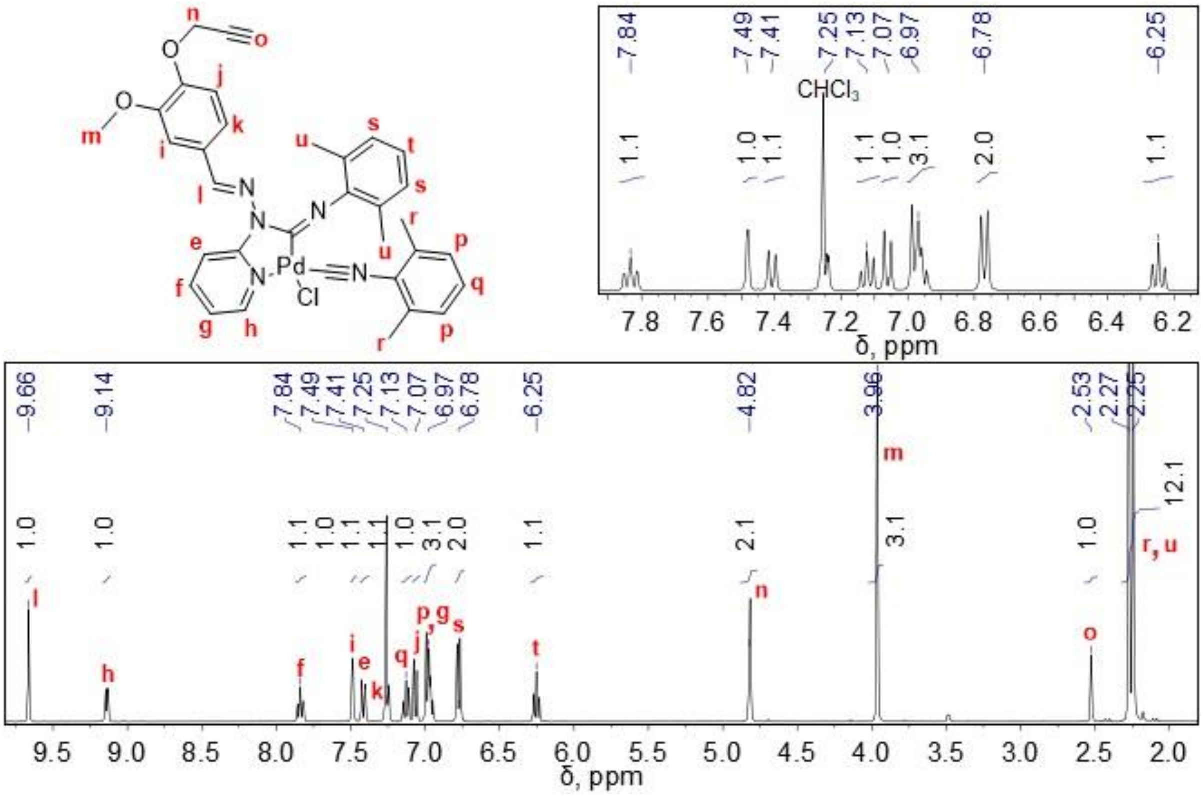

Figure S1. ^{1}H NMR of CMP registered in $CDCl_3$.

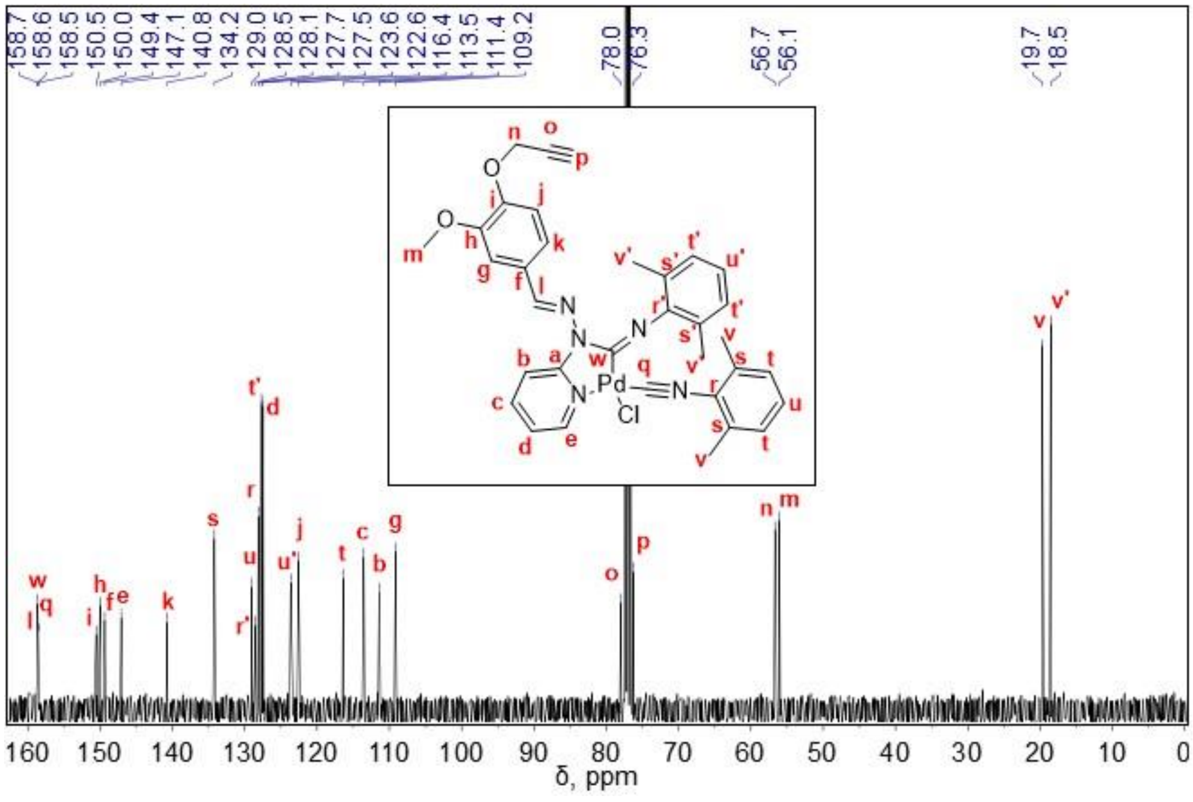

Figure S2. $^{13}C\{^{1}H\}$ NMR of CMP registered in $CDCl_3$.

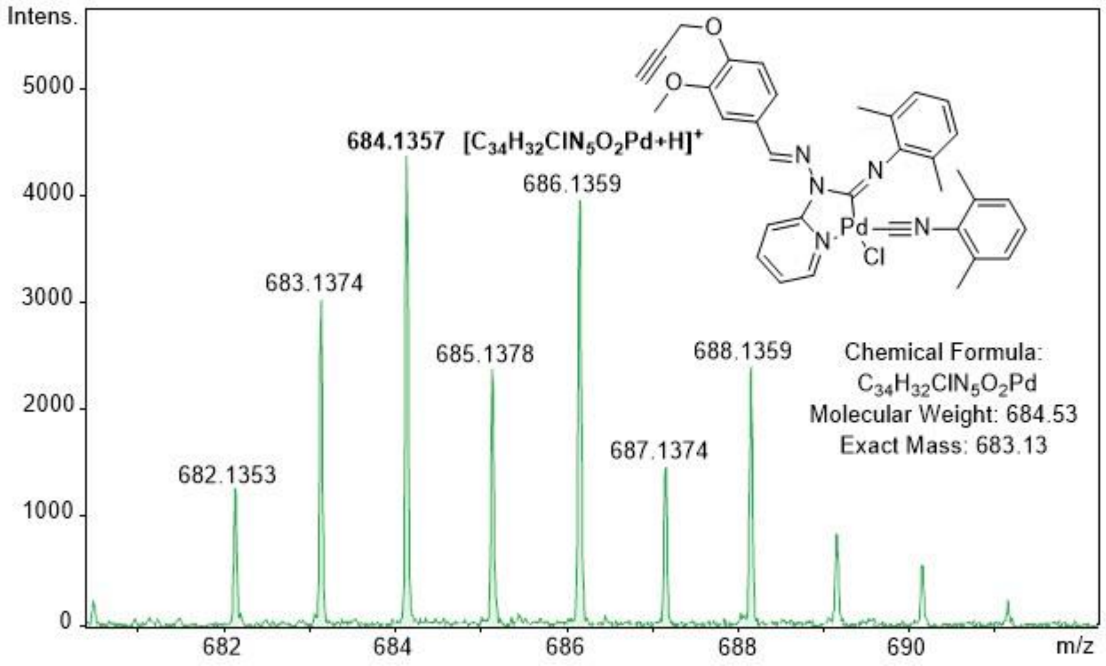

Figure S3. HRESI$^+$ mass spectrum of CMP complex.

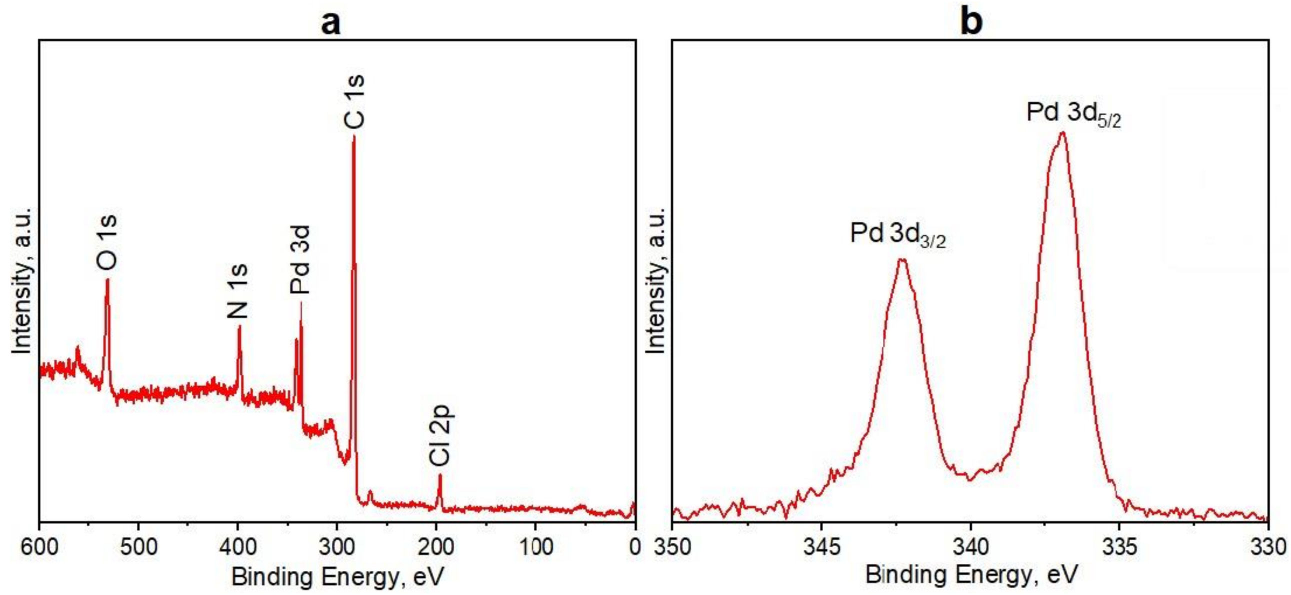

Figure S4.XPS survey spectrum (a) and Pd 3d core level spectrum (b) of CMP.

S2. Catalytic performance of Pd-PDMS

S2.1. ***Reaction conditions for Suzuki–Miyaura reaction in the presence of Pd-PDMS***

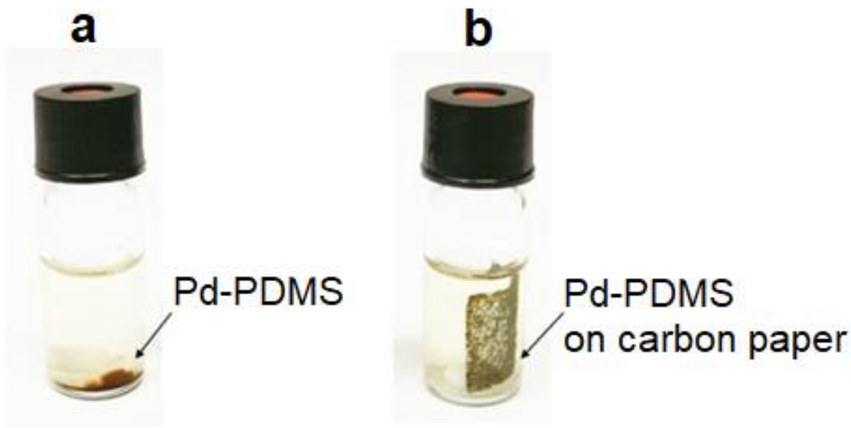

Figure S5. Pd-PDMS applied on walls of a vial (a), Pd-PDMS on carbon paper (CP) (b).

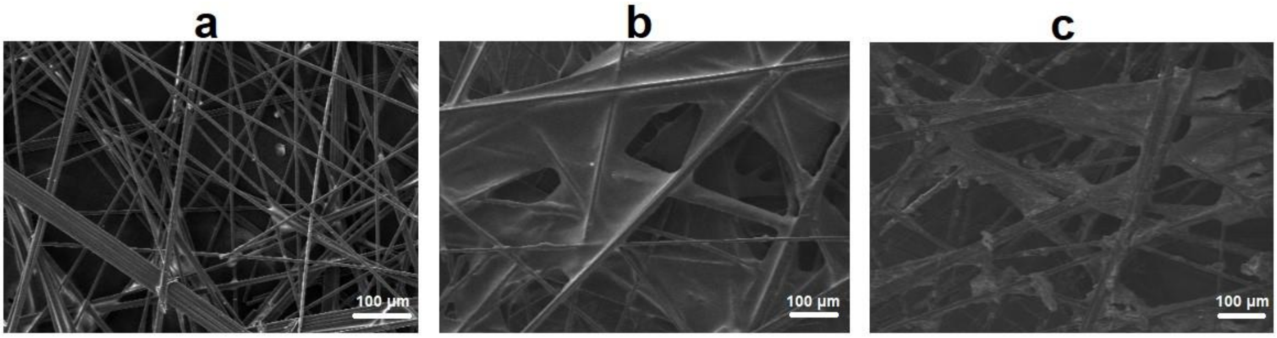
a
b
c
100 µm
100 µm
100 µm

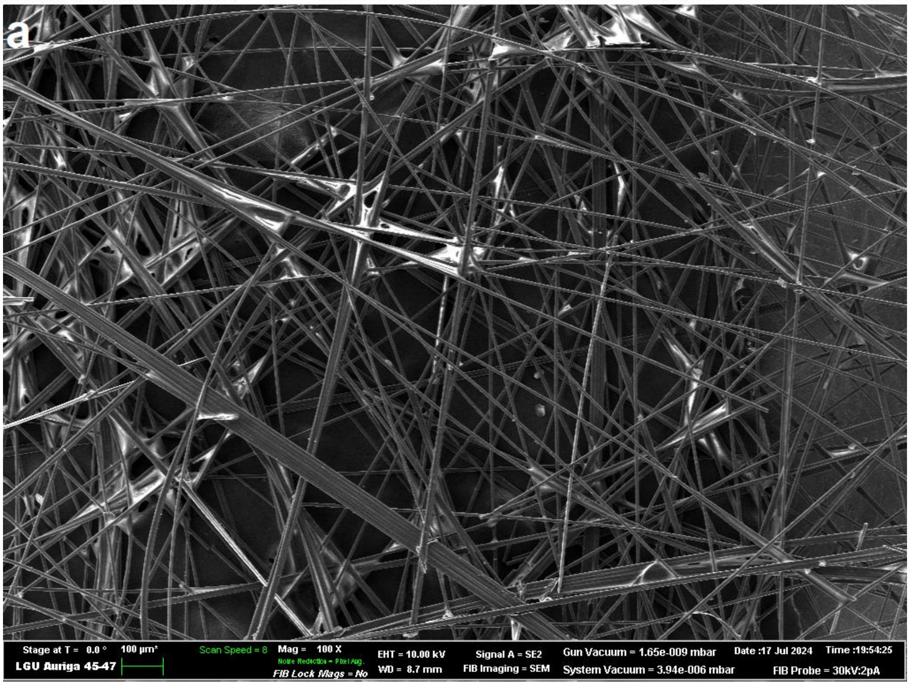
a
Stage at T = 0.0 °
100 µm*
LGU Auriga 45-47
Mag = 100 X
FIB Lock Mags = No
EHT = 10.00 kV
WD = 8.7 mm
Signal A = SE2
FIB Imaging = SEM
Gun Vacuum = 1.65e-009 mbar
System Vacuum = 3.94e-006 mbar
Date :17 Jul 2024
Time :19:54:25
FIB Probe = 30kV:2pA

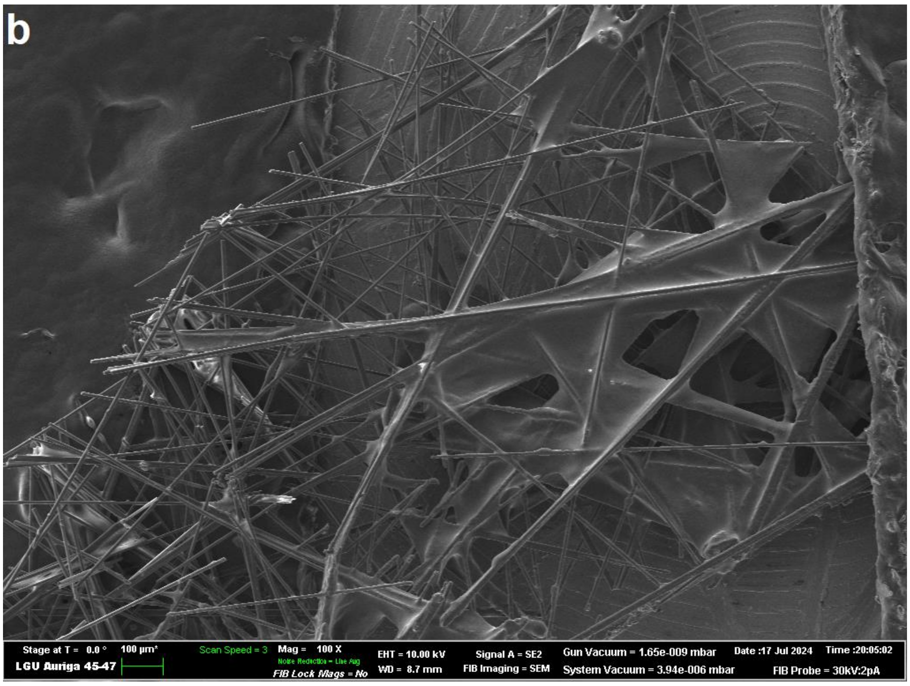
b
Stage at T = 0.0 °
100 µm*
LGU Auriga 45-47
Mag = 100 X
FIB Lock Mags = No
EHT = 10.00 kV
WD = 8.7 mm
Signal A = SE2
FIB Imaging = SEM
Gun Vacuum = 1.65e-009 mbar
System Vacuum = 3.94e-006 mbar
Date :17 Jul 2024
Time :20:05:02
FIB Probe = 30kV:2pA

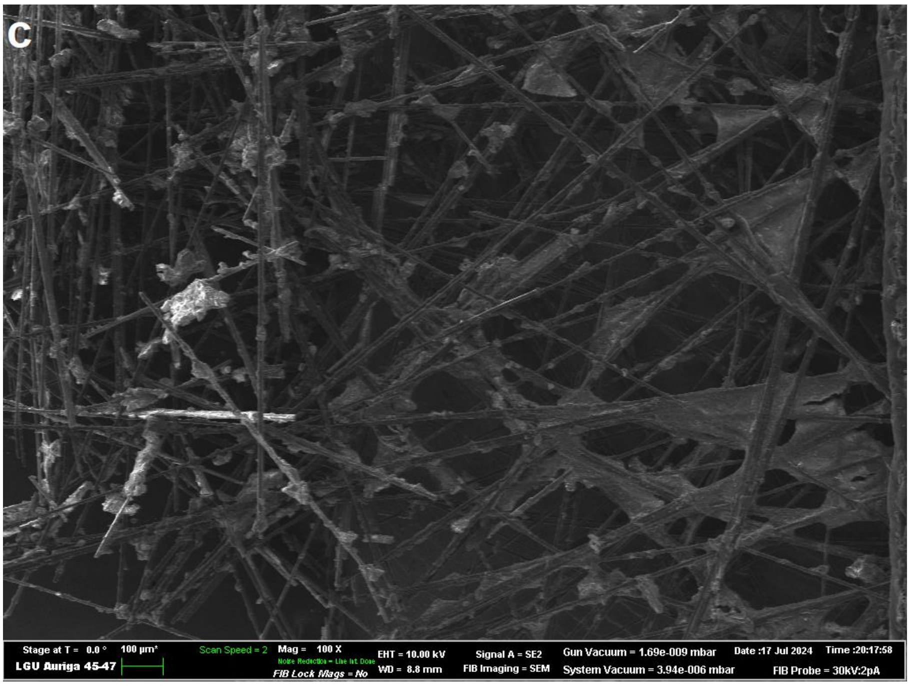

Figure S6. SEM images of pure CP (a), Pd-PDMS on CP before reaction (b) and Pd-PDMS on CP after reaction (c).

S2.2. *Suzuki–Miyaura cross-coupling between 4-bromotoluene and phenylboronic acid*

Prior to use commercially available 4-bromotoluene and phenylboronic acid were analyzed by ^{1}H NMR spectroscopy and their chemical shifts are as follows:

4-bromotoluene ^{1}H NMR ($CDCl_3$, δ): 7.40 (d, 2H, J = 8.2 Hz), 7.07 (d, 2H, J = 8.2 Hz), 2.33 (s, 3H, CH_3).

Phenylboronic acid ^{1}H NMR (DMSO-d_6, δ): 7.99 (s, 2H), 7.78 (dd, 2H, J_1 = 6.6 Hz, J_2 = 1.5 Hz), 7.39 (m, 1H), 7.32 (m, 2H).

To monitor chemical shifts in Suzuki–Miyaura (SM) reaction phenylboronic acid ^{1}H NMR spectrum was also registered in methanol-d_4.

Phenylboronic acid ^{1}H NMR (methanol-d_4, δ): 7.76 (d, 2H, J_1 = 6.6 Hz), 7.36 (m, 4H).

^{1}H NMR data for 4-methylbiphenyl were compared with those reported in literature [1].

4-methylbiphenyl ^{1}H ($CDCl_3$, δ): 2.49 (s, 3H, CH_3), 7.35 (d, J = 8.0 Hz, 2H), 7.37−7.46 (m, 1H), 7.48−7.56 (m, 2H), 7.60 (d, J = 8.1 Hz, 2H), 7.68 (d, J = 7.5 Hz, 2H).

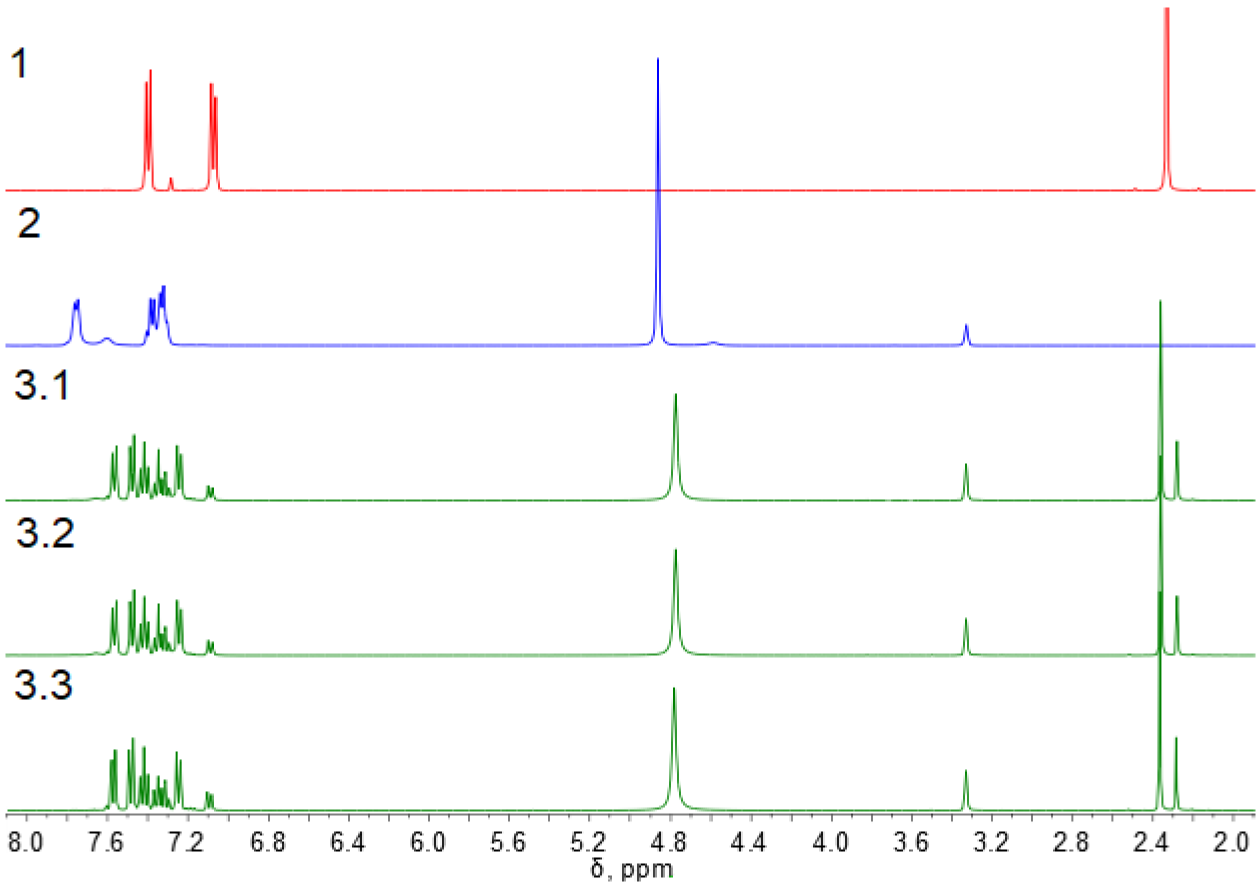

Figure S7. ^{1}H NMR spectra in a range from 1.75 to 8.25 ppm of 4-bromotoluene (1), phenylboronic acid registered in methanol-*d_4* (2), methylbiphenyl 1st catalytic cycle (3.1), methylbiphenyl 2nd catalytic cycle (3.2), methylbiphenyl 3rd catalytic cycle (3.3).

S3. Carbon paper characterization

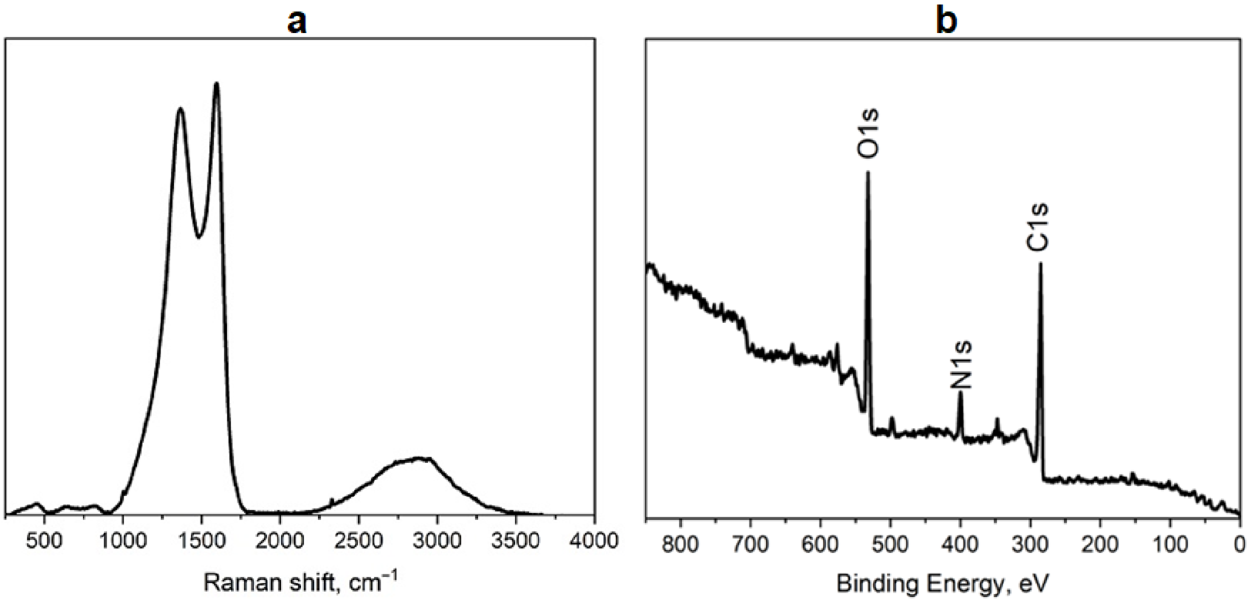

Figure S8. Spectra of the carbon paper used in in catalytic experiments: Raman (**a**) and XPS survey (**b**) spectra.

References:

1. Leadbeater, N.E.; Marco, M. Transition-Metal-Free Suzuki-Type Coupling Reactions. *Angew. Chem. Int. Ed.* **2003**, *42*, 1407–1409, doi:10.1002/anie.200390362.
